# Molecular Divergence upon *EGFR*-TKI Resistance Could Be Dependent on the Exon Location of the Original *EGFR*-Sensitizing Mutation

**DOI:** 10.3390/cancers14184446

**Published:** 2022-09-13

**Authors:** Roberto Serna-Blasco, Estela Sánchez-Herrero, Lucía Robado de Lope, Sandra Sanz-Moreno, Alejandro Rodríguez-Festa, Dunixe Ares-Trotta, Alberto Cruz-Bermúdez, Fabio Franco, Alfredo Sánchez-Hernández, María de Julián Campayo, Carlos García-Girón, Manuel Dómine, Ana Blasco, José M. Sánchez, Juana Oramas, Joaquim Bosch-Barrera, María Á. Sala, María Sereno, Atocha Romero, Mariano Provencio

**Affiliations:** 1 Liquid Biopsy Laboratory, Instituto de Investigación Sanitaria Hospital Puerta de Hierro—Segovia de Arana, 28222 Madrid, Spain; 2 I+D Department, Atrys Health, 08025 Barcelona, Spain; 3 Medical Oncology, Hospital Puerta de Hierro, 28222 Majadahonda, Spain; 4 Medical Oncology, Hospital Provincial Centre de Castelló, 120002 Castellón de La Plana, Spain; 5 Medical Oncology, Hospital Universitario de Burgos, 09006 Burgos, Spain; 6 Medical Oncology, Fundación Jiménez Díaz, 28040 Madrid, Spain; 7 Medical Oncology, Hospital General Universitario Valencia, 46014 Valencia, Spain; 8 Medical Oncology, Hospital de La Princesa, 28006 Madrid, Spain; 9 Medical Oncology, Hospital Universitario de Canarias, 38320 La Laguna, Spain; 10 Medical Oncology, Hospital ICO Girona, 8908 Girona, Spain; 11 Medical Oncology, Hospital Basurto, 48013 Bilbao, Spain; 12 Medical Oncology, Hospital Universitario Infanta Sofía, 28703 San Sebastián de Los Reyes, Spain

**Keywords:** *EGFR*, NGS, *KRAS*, NSCLC, liquid biopsy

## Abstract

**Simple Summary:**

In this study, we report that the exon location of the original *EGFR*-sensitizing mutation could drive resistance mechanisms underlying tumor progression in advanced *EGFR*-positive NSCLC patients under targeted therapies. In our study, plasma detection of the p.T790M *EGFR* resistance mutation, upon disease progression, was more frequent in tumors with an *EGFR* exon 19 deletion (*p* = 0.0028). Furthermore, oncogenic mutations of *KRAS*, arising upon disease progression in 5.6% of the cases, were always detected in patients with tumors harboring *EGFR* exon 18 or 21-sensitizing mutations (*p* < 0.001).

**Abstract:**

Tumor molecular profiling upon disease progression enables investigations of the tumor evolution. Next-generation sequencing (NGS) of liquid biopsies constitutes a noninvasive readily available source of tumor molecular information. In this study, 124 plasma samples from advanced *EGFR*-positive NSCLC patients, treated with a first-line *EGFR* tyrosine kinase inhibitor (*EGFR*-TKI) were collected upon disease progression. The circulating cell-free DNA (cfDNA) was sequenced using the Oncomine Pan-Cancer Cell-Free Assay™. Excluding *EGFR* mutations, the most frequently mutated gene was *TP53* (57.3%), followed by *APC* (11.3%), *FGFR3* (7.3%), and *KRAS* (5.6%). Different molecular alterations were observed upon disease progression depending on the location of the original *EGFR*-sensitizing mutation. Specifically, the detection of the p.T790M mutation was significantly associated with the presence of exon 19 mutations in *EGFR* (Fisher *p*-value: 0.028). All *KRAS* activating mutations (*n* = 8) were detected in tumors with *EGFR* mutations in exons 18 and 21 (Fisher *p*-value < 0.001). Similarly, mutations in *NRAS* and *HRAS* were more frequently detected in samples from tumors harboring mutations in exons 18 or 21 (Fisher *p*-value: 0.050 and Fisher *p*-value: 0.099, respectively). In conclusion, our data suggest that the mechanisms underlying *EGFR*-TKI resistance could be dependent on the exon location of the original *EGFR*-sensitizing mutation.

## 1. Introduction

Lung cancer is currently a serious public health problem. In the United States, the incidence of lung cancer is responsible for approximately one in seven cases of cancer in both sexes, but one in four deaths, being by far the leading cause of cancer death [1]. According to pathology reports, lung cancer can be classified into two main groups: non-small-cell lung cancer (NSCLC) and small-cell lung cancer, accounting for 85% and 15% of the cases, respectively. Among NSCLCs, *EGFR*-positive tumors define a subtype that may benefit from targeted therapies. Overall, *EGFR* mutations are mainly detected in young patients, females, and Asians. The age-standardized incidence rate of *EGFR*-positive NSCLC has been reported to be five per 100,000 person-years [2,3].

Despite *EGFR* inhibitors having dramatically improved the survival outcomes and quality of life of *EGFR*-positive NSCLC patients, drug-resistance mechanisms invariably emerge after treatment, leading to tumor progression within 2 years [4]. Currently, there is intense ongoing research focused on new treatment strategies for *EGFR*-mutated NSCLC patients. As a result, several third-generation *EGFR* tyrosine kinase inhibitors (*EGFR*-TKIs) are in the late stage of clinical development [5,6,7], and fourth-generation *EGFR* inhibitors are being evaluated in preclinical stages and phase I trials [8]. Similarly, the efficacy of the combinations of an *EGFR*-TKI with chemotherapy or antiangiogenic drugs has been evaluated in several trials [9,10]. Other drug combinations such as the third-generation *EGFR*-TKI lazertinib plus amivantamab, a human antibody targeting *EGFR* and *MET*, are under investigation. Furthermore, encouraging results have been reported with more than one-third of *EGFR*-mutant NSCLC patients that progressed on osimertinib showing durable responses [11].

A wide range of survival outcomes is observed in *EGFR*-positive NSCLC. Hence, some *EGFR*-mutant NSCLC patients exhibit a particularly good prognosis with a time to progression exceeding 30 months, while others are diagnosed as having tumor progression within 6 months of *EGFR*-TKI treatment initiation [12]. Heterogeneity caused by different clonal populations may underlie different clinical responses. Indeed, a high clonal diversity has been observed in early-stage *EGFR*-positive NSCLC patients [13,14,15]. In this regard, it was reported that, in advanced *EGFR*-positive NSCLC, tumors harboring concurrent *TP53* or *RB1* mutations showed a higher risk of histologic transformation and inferior sensitivity to *EGFR*-TKI [16,17]. Tumor biopsies in lung cancer upon disease progression are often not feasible or they may not reflect intratumoral heterogeneity and other relevant mutations that may arise in secondary lesions [18,19,20]. In such a scenario, liquid biopsy emerges as an attractive approach for tumor molecular profiling upon disease progression.

In this study, we performed a thorough explorative analysis of *EGFR*-positive NSCLC through NGS profiling of the plasma sample collected upon disease progression of 124 patients in order to characterize the molecular mechanisms via which tumors may progress and to identify the molecular mechanisms underlying different prognoses.

## 2. Materials and Methods

### 2.1. Patients and Samples

A total of 124 patients were recruited by 35 hospitals from February 2016 to September 2021. Written consent was obtained for all enrolled patients. This study was approved by the Ethical Committee of Hospital Puerta de Hierro, Madrid, Spain (internal code: PI 02/16), and conducted in accordance with the precepts of the Code of Ethics of the World Medical Association (Declaration of Helsinki). All patients included in this study were diagnosed with stage IV *EGFR*-positive NSCLC (as per the criteria of the American Joint Committee on Cancer, seventh edition). *EGFR* testing in the FFPE tissue sample was carried out in the pathology department of each participating hospital. Patients were at least 18 years old with a life expectancy of over 12 weeks. All patients were treated with a first-line *EGFR*-TKI. The choice of TKI therapy was left to the discretion of the physician. A plasma sample was collected in an 8.5 mL PPT™ tube (Becton Dickinson, Franklin Lakes, NJ, USA) upon disease progression (*n* = 124). Treatment response was assessed as per Response Evaluation Criteria in Solid Tumors (RECIST) version 1.1 criteria. The average time between the diagnosis disease progression and blood drawn was 5.4 days.

### 2.2. Laboratory Procedures

Blood samples were centrifuged at 1600× *g* for 10 min then immediately at 6000× *g* for 10 min; both centrifugations were performed at room temperature. Hemolyzed samples were excluded. Circulating cell-free DNA (cfDNA) was extracted from the resulting plasma using cfDNA QIAmp Circulating Nucleic Acid kit (Qiagen, Hiden, Germany) following the manufacturer’s protocol. cfDNA was quantified using Qubit 2.0 Fluorometer with a Qubit 1× dsDNA HS Assay Kit (Thermo Fisher, Palo Alto, CA, USA). Libraries were prepared using the Oncomine™ Pan-Cancer Cell-Free Assay kit (Thermo Fisher, Palo Alto, CA, USA), according to the manufacturer’s instructions (the list of genes covered by this panel is available in Supplementary Data S1). The minimum input of cfDNA from each sample required for library preparation was 10 ng, and the maximum volume was 10.4 µL. If less volume of cfDNA was used, RNase-free water was added up to a total volume of 10.4 µL. For library purification, AMPureXP magnetic beads (Beckman Coulter, Inc., Brea, CA, USA) were used. Finally, libraries were diluted to 50 pM using the quantification values obtained from an Ion Library TaqMan^®^ Quantitation Kit (Thermo Fisher, Palo Alto, CA, USA) in a StepOnePlus™ qPCR machine (Thermo Fisher, Palo Alto, CA, USA). Libraries were prepared in batches of eight and were stored at −20 °C up to a maximum of 2 weeks until pool preparation.

A total of 16 samples were sequenced in every NGS run using two Ion 550™ Chips, each of them loaded with eight pooled samples. Templating and Ion 550™ Chip loading were carried out with an Ion Chef™ System (Thermo Fisher, Palo Alto, CA, USA), and then chips were sequenced on an Ion GeneStudio™ S5 Sequencer (Thermo Fisher, Palo Alto, CA, USA). Torrent Suite Software v5.12.2 was used to perform raw sequencing data analysis. The CoverageAnalysis v5.12.2 plugin was used for sequencing coverage analysis. Raw reads were aligned to the human reference genome hg19. Variant calling was carried out on the Ion Reporter platform v.5.18 using Oncomine TagSeq Pan-Cancer Liquid Biopsy—w2.5—Single Sample (workflow versions from w2.1 to w2.5 were used). Variant filtering was performed using an internal pipeline. The pipeline uses the raw data in the non-filtered-oncomine.tsv, which contains variants that have passed the OncomineVariants (v.5.12) filter and variants that have not. Specific conditions were established for single-nucleotide variants (SNVs), insertions or deletions (indels), multiple-nucleotide polymorphisms (MNP), fusions, and copy number variant (CNV) calls. Detailed information about the pipeline is available in Supplementary Figure S1. All candidate mutations were manually reviewed using the Integrative Genomics Viewer (IGV) v.2.3.40, (Broad Institute, Cambridge, MA, USA). The clinical significance of somatic variants was determined according to the Standards and Guidelines for the Interpretation and Reporting of Sequence Variants in Cancer [21]. The pathogenicity of *TP53* and *APC* variants was assessed according to *TP53*-specific ACMG/AMP guidelines [22].

Genomic variants identified by NGS were further confirmed by digital PCR (dPCR) (the mutant allele frequency concordance between NGS and dPCR is shown in Supplementary Figure S2). A complete list of validated variants is available in Supplementary Data S2. In addition, variants identified as potential resistance mechanisms were retrospectively tracked using dPCR (or NGS in the case of CNVs) in all cases in which a baseline sample (before treatment initiation) was available (Supplementary Data S3). dPCR was carried out using predesigned or customized TaqMan^®^ dPCR assays in a QuantStudio^®^ 3D Digital PCR (Applied Biosystems^®^, South San Francisco, CA, USA). An 18 μL final reaction mix was obtained with 8.55 μL of template cfDNA, 9 μL of 20× QuantStudio^®^ Master Mix, and 0.45 μL 40× TaqMan assay. Then, 14.5 μL of the final reaction volume was loaded to a QuantStudio^®^ 3D digital PCR 20K chip. Positive and negative controls were included in every run. Thermal cycler conditions were defined as a first denaturalization step at 96 °C for 10 min, followed by 40 cycles at 56 °C for 2 min and 98 °C for 30 s, an elongation step at 72 °C for 10 min, before finally maintaining the samples at 22 °C for at least 30 min. Then, chip fluorescence was read twice using two independent QuantStudio^®^ 3D Digital PCR instruments. The analysis was performed with QuantStudio^®^ 3D AnalysisSuite™ Cloud; the default call assignment for each read was manually adjusted when needed. The limits of detection and quantitation of the dPCR TaqMan^®^ assays were estimated according to the recommendations of The International Council for Harmonization of Technical Requirements for Pharmaceuticals for Human Use; ICH Q2 (R1) guidelines (validation of analytical procedures: text and methodology), as published elsewhere [23].

### 2.3. Statistical Analysis

Categorical variables are summarized as frequencies, and potential associations were evaluated using the chi-square test or Fisher’s test as appropriate, whereas continuous variables are shown as the mean and standard deviation (non-normally distributed variables are displayed as the median, along with 25th and 75th percentiles), and potential associations were tested using Student’s *t*-test or the Mann–Whitney U test.

Overall survival (OS) was defined as the time from diagnosis of stage IV NSCLC to death from any cause or the censored date of the last follow-up for patients who were alive when the data were extracted. Progression-free survival (PFS) was defined as the time between the start of *EGFR*-TKI treatment and disease progression, as assessed by RECIST criteria, and death from any cause or the censored date of the last follow-up, whichever occurred first. Survival was evaluated using the Kaplan–Meier method with Cox proportional hazards model assumption. The log-rank test was used to assess statistical differences between Kaplan–Meier survival curves. Hazard ratios (HRs) were estimated from the Cox model using a univariate approach. The statistical analysis was performed using R software (v.4.1.2) and Stata version 16.0 (StataCorp 2019, Stata Statistical Software Release 16; StataCorp LLC, College Station, TX, USA).

## 3. Results

### 3.1. Study Cohort

Between February 2016 and September 2021, 124 blood samples from advanced *EGFR*-positive NSCLC patients were collected upon disease progression to a first-line *EGFR*-TKI. The clinical characteristics of the study population are presented in Table 1. Patients were mostly women (77; 62.1%) and never-smokers (71; 57.3%). The mean age at diagnosis was 65.8 years (range 38–89). A total of 116 (93.6%) cases were adenocarcinomas, and the majority were stage IVB (64; 51.6%). Most patients had an Eastern Cooperative Oncology Group Performance Status (ECOG-PS) of 0 or 1 (72; 58.1%). All patients were treated with a first-line *EGFR*-TKI; 62 received afatinib (50%), 26 received erlotinib (21%), 33 received gefitinib (26.6%), and three were treated with osimertinib (2.4%).

According to the pathologist´s report, 68 (54.8%) tumors tested positive for deletions in exon 19, 43 (34.7%) tumors harbored the point mutation p.L858R in exon 21, and six (4.8%) tumors harbored insertions in exon 20. Point mutations at codon 719 (p.G719X), in exon 18, were found in three (2.4%) cases, and the p.L861Q mutation, in exon 21, was also detected in three (2.4%) cases. Lastly, one tumor harbored two concomitant *EGFR*-sensitizing mutations, namely, the p.S768I mutation, in exon 20, and the deletion p.E746_A750del, in exon 19.

Survival data were available for 91 patients. The median follow-up for this population was 46.7 (37.3 to 49.8) months, and the median OS was 23.6 (17.7 to 33) months. Kaplan–Meier curve for PFS, according to the original sensitizing mutations, is depicted in Supplementary Figure S3.

### 3.2. Molecular Landscape upon Disease Progression

**Figure 1:**
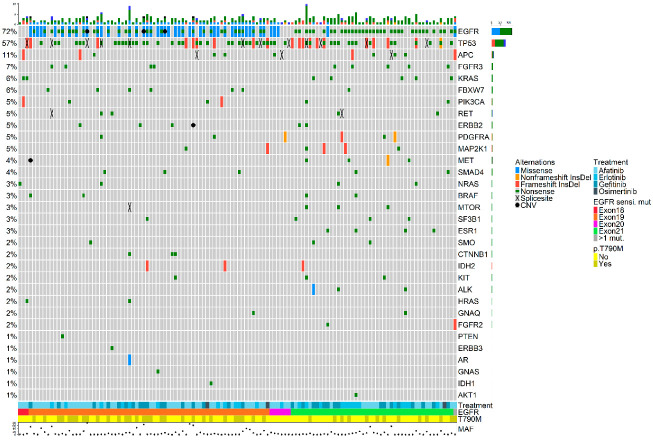
OncoPrint plot showing the distribution of genomic alterations detected in the plasma samples collected upon disease progression. Overview of genomic alterations (legend) in particular genes (rows) for each sample (columns). Missense mutations, non-frameshift deletions or insertions, frameshift deletions or insertions, nonsense mutations, splice site mutations, and CNVs are shown as green rectangles, blue rectangles, red rectangles, black crosses, orange rectangles, and a black dots, respectively. At the bottom of the plot, the following features are presented: first-line *EGFR*-TKI, type of original *EGFR*-sensitizing mutation, and detection of the p.T790M resistance mutation at disease progression. The maximum value of MAF from all detected variants is also shown. Co-occurring mutations were not associated with smoking history and did not have an impact on overall survival (OS). CNV: copy number variant; InsDel: insertion or deletion; MAF: mutated allele frequency.

In total, 365 somatic variants were detected across 32 genes (Figure 1). The most frequent types of variants detected were SNPs (73.9%), followed by indels (24.8%) and CNVs (1.3%). A database containing all detected mutations is available in Supplementary Data S4.

The mean number of detected mutations per sample was 2.9 (range 1–16) with a median mutant allele frequency (MAF) of 5.2% (range 0.1–77.7%) (Supplementary Table S1). Excluding *EGFR* mutations, the most frequently mutated gene was *TP53*, which was mutated in 57.3% of the cases, followed by *APC* (11.3%), *FGFR3* (7.3%), and *KRAS* (5.6%) (Supplementary Table S2). Patients in whom a pathogenic mutation (class 5 or class 4) in *TP53* or *APC* was detected upon disease progression did not show inferior OS compared to patients who did not.

As depicted in Figure 1, a widespread presence of co-occurring genetic alterations was observed. Specifically, 67.7% of the samples had concomitant mutations alongside the original *EGFR*-sensitizing mutation which was detected in the plasma sample of 89 patients (71.8%).

Plasma detection of the original *EGFR*-sensitizing mutation was more challenging in samples from patients with tumor progression exclusively at the brain level, compared with patients diagnosed as having disease progression in other locations (*EGFR* detection rate of 61.5% vs. 76.3%, respectively). On the contrary, the original *EGFR* mutation was detected in all samples from patients with disease progression at the hepatic level (13/13) (*p* = 0.015). The *EGFR* p.T790M resistance mutation was detected in 43 patients.

Oncogenic mutations in other genes were also observed. Specifically, the gain-of-function mutation p.P124L in *MAP2K1* was observed in two cases. Furthermore, the p.E545K mutation in *PIK3CA* was detected in one patient. This mutation was absent at baseline, as well as at 3 months from treatment initiation, further supporting its role as a resistance mutation (Table 2). Similarly, the p.V600E mutation in *BRAF* was detected at disease progression in one case and tested negative in samples collected previously (Table 2; Supplementary Data S3).

Eight oncogenic variants in *KRAS,* namely, p.G12C, p.G12V, p.G12S, p.G12D, p.G15V, p.Q61R, p.Q61H, and p.A146T were detected in seven samples (5.6%) (Table 3). Of them, the p.G12C, p.G15V, p.Q61H, and p.G12D mutations were imputed as acquired resistance mechanisms as they were not detected in the previous samples from the corresponding cases. Conversely, the *KRAS* mutation p.A146T was found at baseline and corresponded to a patient showing the worst PFS (2.8 months) among all *KRAS*-positive cases (Table 2).

**Figure 2:**
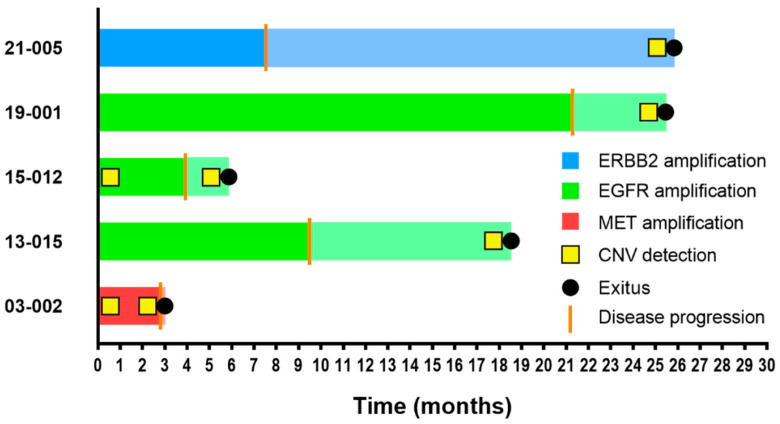
Swimmer plot of PFS and OS in CNV-positive patients. The PFS bar plot appears in dark color, whereas OS is represented continuing the same bar but in attenuated color. Disease progression and exitus events are shown on bar plots as a vertical orange line and a black dot, respectively. CNV detection by NGS is represented with a yellow square.

Lastly, CNVs in *EGFR* (*n* = 3), *MET* (*n* = 1), and *ERBB2* (*n* = 1) were detected in five cases. These CNVs were absent in the pretreatment samples in three cases but were detected at baseline in two cases with *MET* and *EGFR* amplification. Patients in whom CNVs were detected at baseline showed the shortest PFS (2.8 and 3.9 months), suggesting primary resistance through amplification of *MET* or *EGFR,* respectively (Figure 2 and Table S2).

### 3.3. Exon Location of the Original Sensitizing Mutation Determines Distinct Molecular Profiles upon Disease Progression

Detection of the p.T790M mutation was more frequent in tumors harboring an *EGFR* mutation in exon 19 compared with tumors harboring an *EGFR* mutation in exon 21 (*p* = 0.028). Among p.T790M-negative cases, two cases showed clinically actionable mutations upon disease progression, which included a *MET* amplification and the p.G12C mutation in *KRAS*. The druggable p.V600E mutation in *BRAF* was detected in one case, which harbored an exon 19 deletion and tested positive for the p.T790M mutation.

**Figure 3:**
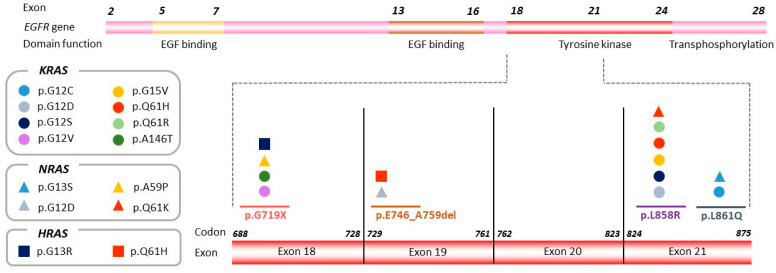
*KRAS, NRAS*, and *HRAS* mutations detected by NGS according to exon location of the original *EGFR*-sensitizing mutation. The *EGFR* gene is represented, and the region including exons 18–21 is magnified in order to show the distribution of *KRAS*, *NRAS*, and *HRAS* variants detected in the plasma sample upon disease progression (represented by circles, rectangles, and squares, respectively), according to exon location of the original *EGFR* sensitizing mutation.

We further tested whether other specific genetic co-alterations tended to be *EGFR* exon-dependent. Comparison of the frequency of genetic co-alterations present in samples from tumors harboring an exon 19 mutation with those with a mutation in exon 21 revealed significant enrichment for *KRAS* mutations in tumors harboring *EGFR*-sensitizing mutations in exons 18 and 21 (*p* < 0.001) (Figure 3). Noteworthy, three of the *KRAS* mutated cases (*n* = 7) harbored an uncommon *EGFR* mutation: the p.G719X mutation in two cases and the p.L861Q mutation in one case. The associations between uncommon *EGFR*-sensitizing mutations and *KRAS* mutations was also significant (*p* = 0.002).

A similar pattern was observed for *NRAS* (*n* = 4) (Supplementary Table S3) and *HRAS* (*n* = 2) (Supplementary Table S4); mutations in these genes were more frequently detected in samples from tumors harboring mutations in exons 18 or 21 (*p* = 0.050 and *p* = 0.099, respectively) (Figure 3).

### 3.4. Prognostic Value of Circulating Tumor DNA

**Figure 4:**
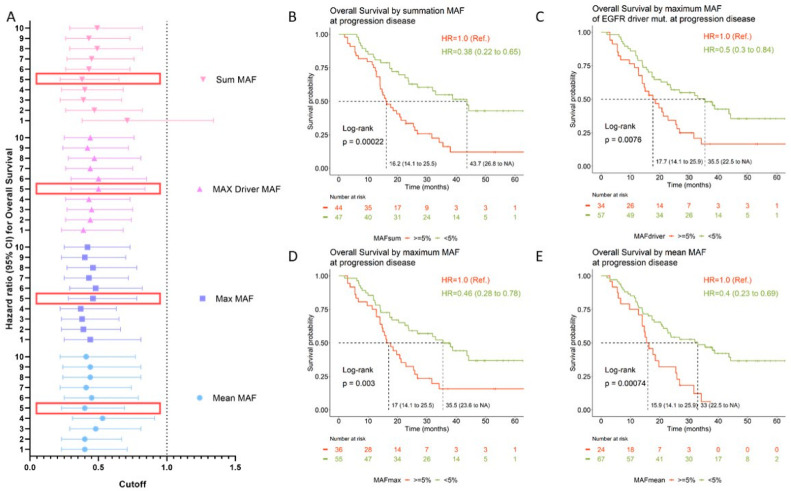
OS according to ctDNA levels upon disease progression. (**A**) Hazard ratios (HRs) for OS are depicted for each MAF cutoff value and according to the quantification approach (mean: blue circles; max: blue squares; max driver: purple triangles; sum: pink upside-down triangles). The horizontal dashed line delimits the confidence intervals. Large red rectangles highlight the MAF cutoff represented in the Kaplan–Meier curves. (**B**–**E**) Kaplan–Meier curves for OS according to MAF cutoff (5%) measured using different methodological approaches. Median OS for each group; log-rank *p*-values and HRs are shown at the left bottom side and right upper side, respectively. Mean MAF was calculated as the mean of the MAF from the set of all variants detected for each sample. Max MAF was defined as the maximum MAF among the set of variants detected for each sample. Driver MAF was defined as the MAF of the *EGFR*-sensitizing mutation. This analysis was carried out using 89 samples which had detectable *EGFR* variants. Sum MAF was calculated as the sum of MAF from the set of variants detected for each sample. CI: confidence interval; CNV: copy number variant; HR: hazard ratio; MAF: mutant allele frequency; NA: not available; Ref.: reference category.

The prognostic value of circulating tumor DNA (ctDNA) levels was evaluated using different methodological approaches. To this aim, we calculated the mean, maximum, and sum of MAF from the set of all detected variants for each sample, and we established different MAF thresholds (ranging from 1% to 10%). In addition, the prognostic value of the MAF of the original *EGFR*-sensitizing mutation was tested. As presented in Table 4 and Figure 4A, the amount of ctDNA was of prognostic significance regardless of the approach used. Overall, patients with high ctDNA at disease progression had significantly worse OS than those patients in which the opposite situation occurred (Table 4 and Figure 4A). Kaplan–Meier curves for the <5%MAF cutoff are shown in Figure 4B–E. Using a cutoff of sum MAF < 5%, the median OS was 16 months (95% CI: 14 to 21.2) in patients with high ctDNA levels (MAF ≥ 5%) compared with 43.7 months (95% CI: 23.6 to NR) for patients with ctDNA levels below that cutoff.

## 4. Discussion

Several different mechanisms of acquired resistance to *EGFR* inhibitors have been described so far [24,25,26]. The wide variety of resistance mutations highlights the importance of tumor heterogeneity in shaping tumor resistance to targeted therapies. To our knowledge, here, we report for the first time that *EGFR*-TKI resistance through acquired *KRAS* mutation could be dependent on the exon location or type of mutation of the original *EGFR*-sensitizing mutation, suggesting that tumor resistance could be driven by the position in the genome of the original *EGFR* mutation. This hypothesis is plausible considering that it is well established that *EGFR*-mutant NSCLC patients have different sensitivity to targeted therapies according to the exon in which the original sensitizing mutation is detected [27]. This circumstance was also observed in our cohort (Supplementary Figure S3). In other words, *EGFR*-positive NSCLC tumors can be classified into different subtypes with different survival outcomes defined by the location of the *EGFR*-sensitizing mutation. In this way, unlike *EGFR* exon 19 deletions and point mutations in exon 21, most NSCLC tumors harboring *EGFR* exon 20 insertion mutations do not benefit from *EGFR*-TKIs with response rates reported to be below 5% and short intervals of disease control [28]. Indeed, important efforts have been made in order to develop effective therapies for this particular subset of patients in recent years [29].

Overall, TKI resistance can be classified into *EGFR*-dependent and *EGFR*-independent mechanisms. The p.T790M mutation is the most commonly observed resistance mechanism in NSCLC patients treated with first- and second-generation *EGFR*-TKIs [30,31,32]. In our study, the p.T790M mutation was significantly more frequently detected in tumors harboring a mutation in exon 19. This finding is consistent with previous reports [12,33]. Furthermore, in our cohort, amplifications in *EGFR*, *MET*, and *ERBB2* were detected in five cases. *MET* gene amplification has been identified as a resistance mechanism for afatinib, gefitinb, erlotinib, and osimertinib [34,35,36,37]. Indeed, dual inhibition of *EGFR* and *MET* represents a promising treatment strategy [11]. Similarly, *ERBB2* amplifications have been observed in tumors with acquired resistance to erlotinib, gefitinib, and osimertinib [38,39]. Oncogenic mutations in *MAP2K1* and *PIK3CA* genes were also found in three cases. It is well established that activation of downstream *EGFR* signaling pathways such as *MAPK*/*ERK* or *PIK3CA*/*AKT* signaling pathways play an important role in *EGFR*-TKI resistance [40,41]. Similarly, acquired mutations in *BRAF* have been shown to underline *EGFR*-TKI resistance [42,43]. In our cohort, the p.V600E mutation in *BRAF* was detected alongside the p.T790M resistance mutation in one patient whose tumor harbored a deletion in exon 19. Similarly, a case report by Chao-Chi Ho et al. reported the acquisition of the mutation *BRAF* p.V600E in a patient with p.T790M at the time of progression while being treated with osimertinib [42]. In our study, ctDNA profiling was carried out with a relatively small NGS panel. The Oncomine™ Pan-Cancer Cell-Free Assay kit (Thermo Fisher, Palo Alto, CA, USA) covers hotspots in 52 genes. Therefore, these results should be interpreted with caution and they must be validated with larger cohorts.

The fact that *KRAS* mutations were more frequently detected in tumors harboring mutations in exon 18 and 21 or uncommon mutations may have important implications for the development of clinical trials evaluating the efficacy of dual or consecutive *EGFR* and *KRAS* blockage. It has previously been documented that the druggable mutation *KRAS* p.G12C is found in approximately 1% of *EGFR*-positive NSCLC patients progressing on a first-line treatment with an *EGFR*-TKI, and it tended to arise in tumors harboring *EGFR* uncommon mutations [44], supporting our findings.

Lastly, the amount of ctDNA at disease progression significantly correlated with OS. There is large evidence indicating that ctDNA levels significantly correlate with tumor bulk and, therefore, can be used to monitor disease [12,45,46]. Moreover, ctDNA levels are of prognostic significance [12]. Indeed it has been proposed to include ctDNA in the tumor staging system [47]. Nevertheless, it is not well established how ctDNA should be measured, especially when tumors do not harbor druggable mutations. As an exploratory approach, here, we evaluated the prognostic value using different methods (median of MAF from all mutations detected, maximum MAF of all detected mutation, MAF of the original *EGFR*-sensitizing mutation, and summation MAF of all detected mutations), and similar results were obtained.

## 5. Conclusions

Different molecular heterogeneous alterations were observed upon disease progression, highlighting the importance of heterogeneity driving tumor resistance. Our data suggest that the mechanisms underlying resistance could be dependent on the exon location of the original *EGFR*-sensitizing mutation. Further studies are warranted to confirm this observation.

## Figures and Tables

**Table 1:** Descriptive analysis of the study cohort.

Clinicopathologic Characteristics	*N* = 124
Age, mean (SD), years	65.8 (11.1)
Sex, No. (%) with data	
Female	77 (62.1)
Male	47 (37.9)
Smoking, No. (%) with data	
Never-smokers	71 (57.3)
Active smokers	9 (7.2)
Former smokers	44 (35.5)
ECOG-PS ^a^, No. (%) with data	
0	35 (28.2)
1	37 (29.8)
2	5 (4.0)
Stage, No. (%) with data	
IVA	60 (48.4)
IVB	64 (51.6)
Histology, No. (%) with data	
Adenocarcinoma	116 (93.6)
Adenosquamous	2 (1.6)
Large cell	4 (3.2)
Squamous	2 (1.6)
Metastases at IV stage diagnosis ^b^, No. (%) with data	
Local	88 (71)
Bone	61 (49.2)
CNS	18 (14.5)
Liver	17 (13.7)
Progression site ^c^, No. (%) with data	
Bone	21 (16.9)
CNS	13 (10.5)
Liver	13 (10.5)
*EGFR* mutation, No. (%) with data	
Del19	68 (54.8)
G719X	3 (2.4)
Ins20	6 (4.8)
L858R	43 (34.7)
L861Q	3 (2.4)
>1 mut.	1 (0.8)
Treatment, No. (%) with data	
Afatinib	62 (50)
Erlotinib	26 (21)
Gefitinib	33 (26.6)
Osimertinib	3 (2.4)
Second line treatment ^d^, No. (%) with data	
Osimertinib	63 (50.8)
Others	25 (20.2)
None	8 (6.4)
Exitus ^e^	
Yes	59 (47.6)
No	32 (25.6)

^a^ 47 patients without information; ^b^ 5 patients without information; ^c^ 52 patients without information; ^d^ 28 patients without information; ^e^ 33 patients without information. Abbreviations: CNS: central nervous system; ECOG-PS: Eastern Cooperative Oncology Group Performance Status.

**Table 2:** dPCR Validation and retrospective tracking of oncogenic mutations detected upon disease progression.

Sample	Gene	Coding Transcript Change	Protein Change	Functional Classification	PFS ^b^	OS ^b^	B	≈3	≈6	P
400003	*KRAS*	c.35G>T	p.G12V	Missense	5.8	NA	-	-	-	●
1000040	*KRAS*	c.182A>G	p.Q61R	Missense	9.7	NA	-	-	-	●
8500002	*KRAS*	c.34G>A	p.G12S	Missense	12.3	NA	-	-	-	●
03-002 ^a^	*MET*	CNV	CNV	CNV	2.8	3	●			●
11-006	*KRAS*	c.34G>T	p.G12C	Missense	11.7	15.5	●	●	●	●
11-008	*PIK3CA*	c.1633G>A	p.E545K	Missense	7.7	15	●	●	-	●
13-014	*BRAF*	c.1799T>A	p.V600E	Missense	15.8	34.2	●	●	●	●
13-015 ^a^	*EGFR*	CNV	CNV	CNV	9.5	18.5	●			●
15-012 ^a^	*EGFR*	CNV	CNV	CNV	3.9	5.9	●			●
19-001 ^a^	*EGFR*	CNV	CNV	CNV	21.3	25.5	●			●
21-005 ^a^	*ERBB2*	CNV	CNV	CNV	7.5	25.9	●			●
26-004	*KRAS*	c.436G>A	p.A146T	Missense	2.8	14.3	●	-	-	●
33-001	*KRAS*	c.44G>T	p.G15V	Missense	11.9	30.4	●	●	●	●
36-005	*KRAS*	c.183A>C	p.Q61H	Missense	7.6	9.7	●	●	●	●
36-005	*KRAS*	c.35G>A	p.G12D	Missense	7.6	9.7	●	●	●	●

^a^ Samples from this patient were evaluated by NGS. ^b^ Calculated in months. Red dots represent that the variant was absent in the sample, green dots indicate detection of the mutation in the sample, and yellow dots are depicted when the variant could not be validated by dPCR due to the lack of sample. In this case, only the NGS data are available. Three *KRAS* variants (p.G15V, p.Q61H, and p.Q61R) were not confirmed by dPCR because of a lack of sample, whereas the remaining *KRAS* mutations were validated by dPCR. Unfortunately, there were no previous samples for the patients whose tumors harbored p.G12V, p.Q61R, and G12S mutations. Abbreviations: B: baseline sample (before the start of treatment); ≈3: sample extracted during treatment (after 3 months of treatment approximately); ≈6: sample extracted during treatment (after 6 months of treatment approximately); P: sample extracted at the moment of disease progression; PFS: progression-free survival; OS; overall survival.

**Table 3:** Clinicopathological features of *KRAS* mutated cases.

	400003	1000040	8500002	11-006	26-004	33-001	36-005
***KRAS* mutation by NGS (coding/protein)**	c.35G>T/p.G12V	c.182A>G/p.Q61R	c.34G>A/p.G12S	c.34G>T/p.G12C	c.436G>A/p.A146T	c.44G>T/p.G15V	c.183A>C/p.Q61H c.35G>A/p.G12D
**MAF *KRAS* mutation (%)**	4.15	0.18	0.62	18.11	0.19	0.57	3.67/0.64
***EGFR* mutation by NGS (coding/protein)**	Not detected	c.2573T>G/p.L858R	c.2573T>G/p.L858R	c.2582T>A/p.L861Q	Not detected	Not detected	c.2573T>G/p.L858R
**MAF *EGFR* mutation (%)**	-	0.30	9.77	2.15	-	-	25.63
**p.T790M by NGS**	Not detected	Not detected	Detected	Not detected	Not detected	Not detected	Not detected
***EGFR* mutation in tumor at stage IV diagnosis**	p.G719X	p.L858R	p.L858R	p.L861Q	p.G719X	p.L858R	p.L858R
**Sex**	Male	Female	Male	Female	Female	Female	Female
**Age (years)**	74	76	74	68	58	79	52
**Smoking**	Former smoker	Former smoker	Former smoker	Smoker	Never-smoker	Never-smoker	Never-smoker
**Histology**	Adenocar.	Adenocar.	Adenocar.	Adenocar.	Adenocar.	Adenocar.	Adenocar.
**ECOG-PS**	NA	NA	NA	0	1	1	1
**Metastasis location at stage IV diagnosis**	Thoracic and bone	Bone	Thoracic	Thoracic	Bone and CNS	Thoracic	CNS
**Stage**	IVB	IVB	IVA	IVA	IVB	IVA	IVB
**First-line TKI**	Afatinib	Gefitinib	Afatinib	Afatinib	Afatinib	Erlotinib	Afatinib
**PFS (months)**	5.8	9.7	12.3	11.7	2.8	11.9	7.6
**Progression site**	NA	NA	NA	CNS	Liver	NA	NA
**Second-line treatment**	None	Gefitinib	Osimertinib	None	None	Chemoth.	Osimertinib
**Exitus**	NA	NA	NA	Yes	Yes	No	Yes
**OS (months)**	NA	NA	NA	15.5	14.3	30.4	9.7

Adenocar.: adenocarcinoma; Chemoth.: chemotherapy; CNS: central nervous system; ECOG-PS: Eastern Cooperative Oncology Group Performance Status; MAF: mutant allele frequency; NA: not available; NGS: next-generation sequencing; OS: overall survival; PFS: progression-free survival.

**Table 4:** HR and corresponding 95% CI according to MAF cutoff assessed using four different approaches.

MAF Cutoff	Mean MAF ^a^		Max MAF ^b^		Driver MAF ^c^		Sum MAF ^d^	
	HR (95%CI)	*p*-Value	HR (95%CI)	*p*-Value	HR (95%CI)	*p*-Value	HR (95%CI)	*p*-Value
1%	0.40 (0.23–0.71)	0.002	0.44 (0.25–0.81)	0.008	0.39 (0.23–0.68)	0.001	0.71 (0.38–1.34)	0.295
2%	0.40 (0.23–0.67)	0.001	0.39 (0.23–0.66)	0.001	0.44 (0.26–0.74)	0.002	0.47 (0.26–0.82)	0.009
3%	0.48 (0.29–0.81)	0.006	0.38 (0.23–0.65)	<0.001	0.45 (0.27–0.75)	0.003	0.39 (0.22–0.67)	0.001
4%	0.53 (0.31–0.91)	0.022	0.37 (0.22–0.63)	<0.001	0.43 (0.26–0.73)	0.002	0.40 (0.23–0.68)	0.001
5%	0.40 (0.23–0.69)	0.001	0.46 (0.28–0.78)	0.004	0.50 (0.30–0.84)	0.009	0.38 (0.22–0.65)	<0.001
6%	0.45 (0.25–0.79)	0.006	0.48 (0.29–0.82)	0.007	0.50 (0.30–0.85)	0.010	0.43 (0.26–0.73)	0.002
7%	0.41 (0.22–0.74)	0.003	0.43 (0.25–0.72)	0.002	0.44 (0.26–0.75)	0.003	0.45 (0.27–0.76)	0.003
8%	0.44 (0.24–0.81)	0.008	0.46 (0.27–0.78)	0.004	0.47 (0.28–0.81)	0.007	0.49 (0.29–0.82)	0.006
9%	0.44 (0.24–0.81)	0.008	0.40 (0.23–0.70)	0.001	0.42 (0.24–0.72)	0.002	0.43 (0.26–0.73)	0.002
10%	0.41 (0.22–0.77)	0.005	0.42 (0.25–0.73)	0.002	0.44 (0.25–0.76)	0.003	0.49 (0.29–0.82)	0.007

^a^ Mean MAF was calculated as the mean of the MAF set of variants detected for each sample. ^b^ Max MAF was defined as the maximum MAF among the set of variants detected for each sample. ^c^ Driver MAF was defined as the maximum MAF among *EGFR*-sensitizing variants detected for each sample. This analysis was carried out using 89 samples which had detectable *EGFR* variants. ^d^ Sum MAF was calculated as the summation of the MAF set of variants detected for each sample. HRs and *p*-values were calculated using univariate Cox model analysis. All data were estimated for overall survival. CI: confidence interval; HR: hazard ratio; MAF: mutant allele frequency.

## Data Availability

In this study, an internal bioinformatics pipeline, designed using R, was used. A schematic flowchart of the pipeline is shown in Supplementary Figure S1. The data presented in this study are available on request from the corresponding author.

## Supplementary Materials

The following are available online at https://www.mdpi.com/article/10.3390/cancers14184446/s1, Figure S1: Flowchart of the bioinformatic pipeline optimized for processing and assessing variants using the Oncomine™ Pan-Cancer Cell-Free Assay; Figure S2: Linear correlation between MAF assessed by NGS and dPCR for driver EGFR and p.T790M mutations; Figure S3: Kaplan–Meier curve for PFS according to exon location of EGFR-sensitizing mutation at diagnosis of advanced NSCLC; Table S1: Types of mutations detected using NGS. MAF and MAPD parameters; Table S2: Number of patients with tumors harboring mutations in each gene covered by the NGS panel; Table S3: Relevant information regarding *NRAS*-mutated patients; Table S4: Relevant information regarding *HRAS*-mutated patients; Data S1: Datasheet with a list of genes included in the Oncomine™ Pan-Cancer Cell-Free Assay kit (Thermo Fisher, Palo Alto, CA, USA) (Format: .xlsx); Data S2: Datasheet with all TaqMan^®^ dPCR assays (predesigned or customized) (Thermo Fisher, Palo Alto, CA, USA) available in our laboratory (format: .xlsx); Data S3: Datasheet with variants considered as potential mechanism of resistance with information about validation by dPCR/NGS and its detection in previous samples (format: .xlsx); Data S4: Final list of the genetic variants (*N* = 365) found through NGS for this study (format: .xlsx).

## Abbreviations

cfDNA, circulating cell-free DNA; CI, confidence interval; CNS, central nervous system; CNV, copy number variant; ctDNA, circulating tumor DNA; dPCR, digital PCR; ECOG eastern cooperative oncology group; *EGFR*-TKI, *EGFR* tyrosine kinase inhibitor; HR, hazard ratio; IGV, integrative genome visualizer; Indel, insertion or deletion; MAF, mutated allele frequency; MNP, multiple-nucleotide polymorphism; NGS, next-generation sequencing; NR, not reached; NSCLC, non-small-cell lung cancer; OS, overall survival; PFS, progression-free survival; RECIST, Response Evaluation Criteria in Solid Tumors; SNV, single-nucleotide variant.
