# Supplementary material for: Molecular Divergence upon EGFR-TKI Resistance Could Be Dependent on the Exon Location of the Original EGFR-Sensitizing Mutation
Source: Cancers (Basel). 2022 Sep 13;14(18):4446. doi: 10.3390/cancers14184446 (PMC9496947; doi:10.3390/cancers14184446)
Supplement: Supplementary file 1 [file cancers-14-04446-s001.zip › Supplementary_Figures.pdf]

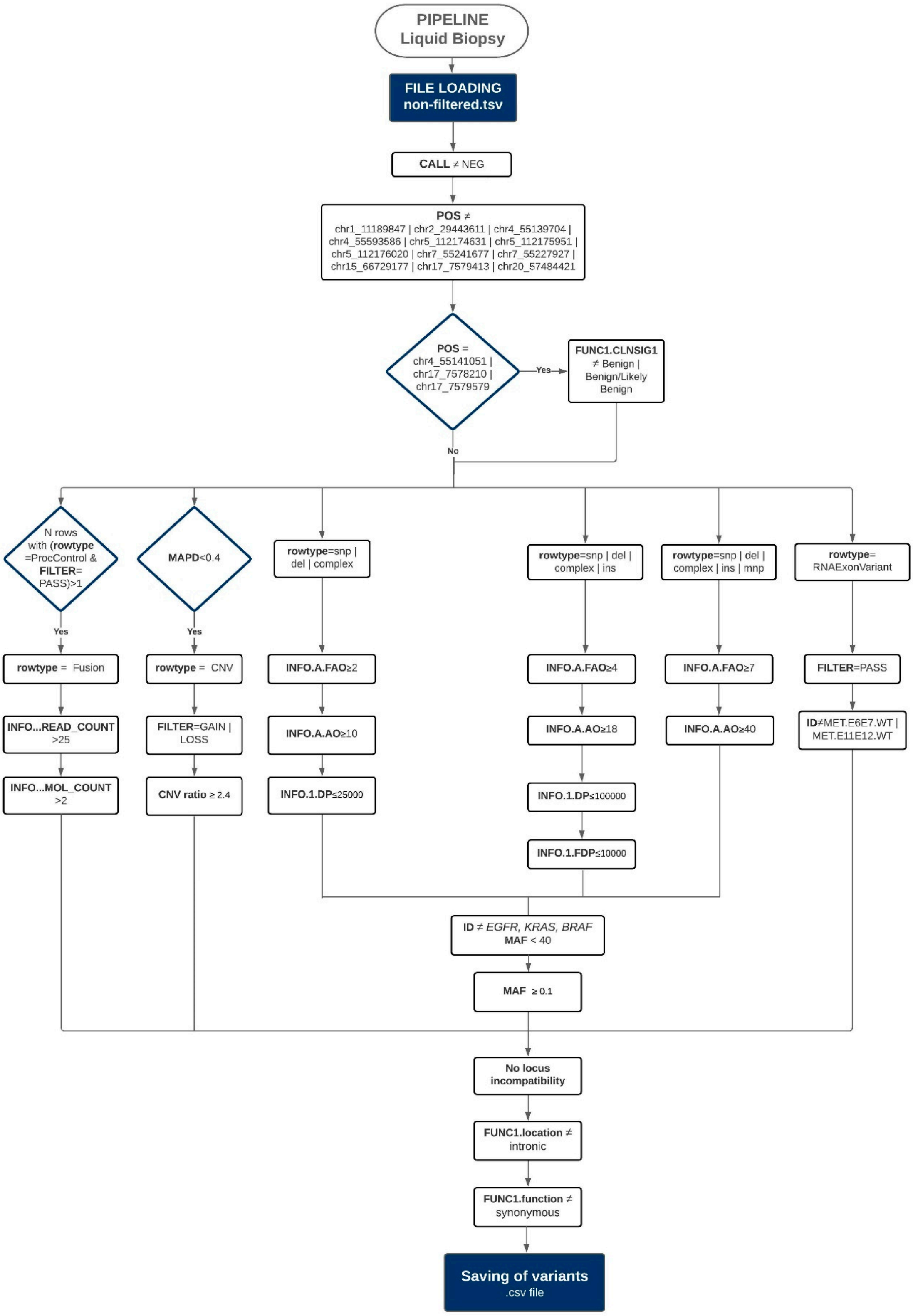

Supplementary Figure S1. Flowchart of the bioinformatic pipeline optimized for processing and assessing variants using the Oncomine™ Pan-Cancer Cell-Free Assay.

The non-filtered-oncomine.tsv file was obtained for all the samples. Mutations with CALL attribute equal to NEG were discarded. Variants located in 'noisy-regions' (genome positions: 11189847, 29443611, 55139704, 55593586, 112174631, 112175951, 112176020, 55241677, 55227927, 66729177, 7579413 and 57484421 on chromosomes 1, 2, 4, 4, 5, 5, 5, 7,

7, 15, 17 and 20 respectively) were filtered out. In addition, when variants were located at the following genome positions: chr_455141051, chr7_7578210, chr7_7579579 and they were classified as benign or likely-benign, were excluded.

Different conditions were established according to variant type as follows:

Fusions: The panel includes two control target genes, namely TBP and HMBS. Both controls must have a molecular count (MC) >2 to pass quality control (ROW_TYPE= "ProcControl"; FILTER= "PASS"). Fusions must have >2 MC and >25 molecular reads (MR) to be reported (branch 1).

Copy number variant (CNV): For a CNV call the MAPD (Median of the Absolute values of all Pairwise Differences) must be <0.4. MAPD is a quality metric that estimates coverage variability between adjacent amplicons in CNV analyses. The higher MAPD the lower coverage uniformity, resulting in a higher probability of erroneous CNV calls. CNV Ratio for a copy number gain must be >2.4 (branch 2).

False positives are less likely in SNPs and very likely in MNPs. For this reason, different conditions were established according to variant type.

Single nucleotide polymorphism (SNP): the algorithm makes a positive call as long as any of the following conditions are met: SNPs have to be detected in at least two MC with >10 MR and ≤ 25000 global reads (branch 3). SNPs have to be detected in at least four MC with >18 MR, ≤ 100000 global reads, and ≤ 10000 global MC (branch 4). SNPs have to be detected in at least seven MC with >40 MR (branch 5).

Deletions (del): For a variant call the same conditions as SNPs selected via branch 3, 4 and 5 are required.

Complex: Complex variant selection follows the same conditions as SNPs selected via branch 3, 4 and 5.

Insertions (ins): Insertion selection follows the same conditions as SNPs selected via branch 4 and 5.

Multiple nucleotide polymorphism (MNP): MNPs were only selected if they were detected in at least seven MC and ≥40 MR (branch 5).

RNA Exon Variants: Panel includes two *MET* wt control amplicons. At least 1 of these controls must have a MC >2 to pass quality control. Exon Skipping amplicons must have >2 MC to be reported (branch 6).

SNPs, del, ins and MNPs must have a mutated allele frecuency (MAF) equal or higher than 0.1% and lower than 40% (except for *EGFR*, *KRAS* and *BRAF* genes). Finally, all selected variants were saved whenever they meet the following conditions; no locus incompatibility, variant location has to be other than intronic and function variant has to be no synonymous.

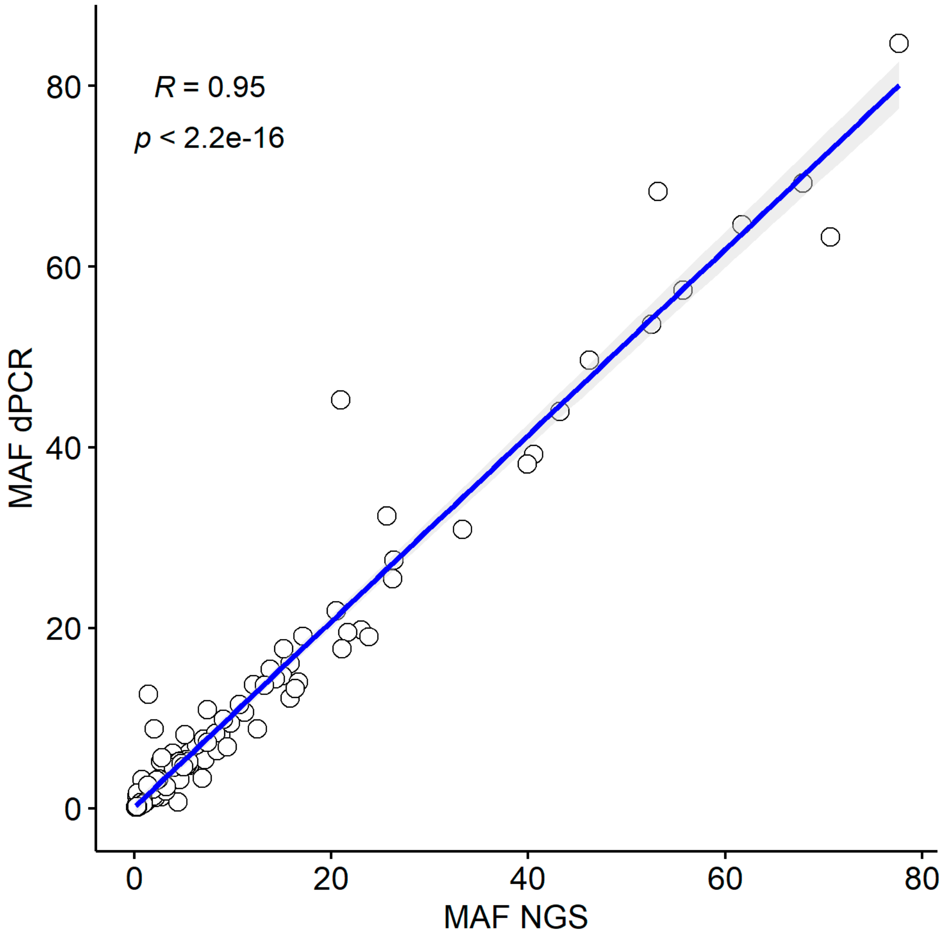

Supplementary Figure S2. Linear correlation between MAF assessed by NGS and dPCR for driver *EGFR* and p.T790M mutations.

Linear regression function is represented in blue, and the 95% confidence interval is shaded in grey. Spearman's coefficient and the corresponding p-value are shown in the plot. MAF: mutated allele frequency.

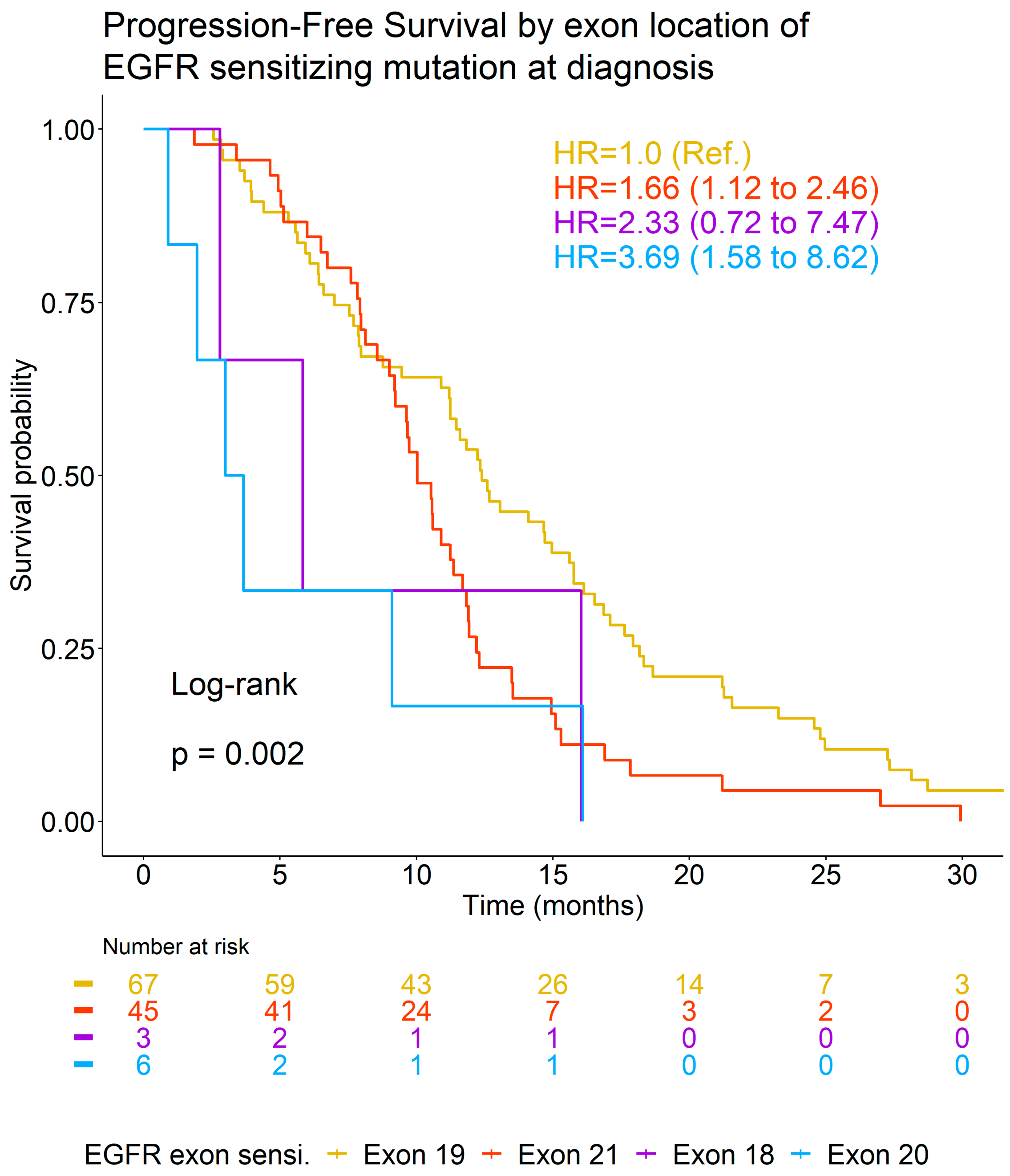

Supplementary Figure S3. Kaplan-Meier curve for PFS according exon location of *EGFR* sensitizing mutation at diagnosis of advanced NSCLC. Hazard ratios with corresponding 95% CI from Cox-Model and p-value from logrank test are shown. HR: Hazard ratio. Ref.: Reference category. Sensi.: Sensitizing.
