# Supplementary material for: Molecular Divergence upon EGFR-TKI Resistance Could Be Dependent on the Exon Location of the Original EGFR-Sensitizing Mutation
Source: Cancers (Basel). 2022 Sep 13;14(18):4446. doi: 10.3390/cancers14184446 (PMC9496947; doi:10.3390/cancers14184446)
Supplement: Supplementary file 1 [file cancers-14-04446-s001.zip › Supplementary_Tables.pdf]

Supplementary Table S1. Type of mutations detected by NGS. MAF and MAPD parameters.

Mutations	N=365
Mean per sample	2.9 (1; 16)
Mutation type, No. (%) with data	
snp	264 (72.3)
insdel	96 (26.3)
cnv	5 (1.4)
Functional classification, No. (%) with data	
CNV	5 (1.4)
Frameshift deletion	20 (5.5)
Frameshift insertion	15 (4.1)
Missense	250 (68.5)
Nonframeshift block substitution	4 (1.1)
Nonframeshift deletion	44 (12)
Nonframeshift insertion	3 (0.8)
Nonsense	20 (5.5)
unknown	4 (1.1)
Location, No. (%) with data	
Exonic	356 (98.9)
Splice site 3′	2 (0.6)
Splice site 5′	1 (0.3)
UTR 5′	1 (0.3)
MAF, mean (min; max), %	5.2 (0.1; 77.7)
MAPD, mean (min; max), %	0.3 (0.1; 1.8)

MAF: Mutant allele frequency; MAPD: Median of the absolute values of all pairwise differences.

Supplementary Table S2. Number of patients with tumors harboring mutations in each gene covered by the NGS panel.

Gene	N patients	Frequency (%)
EGFR	89	71,8
TP53	71	57,3
APC	14	11,3
FGFR3	9	7,3
FBXW7	7	5,6
KRAS	7	5,6
ERBB2	6	4,8
MAP2K1	6	4,8
PDGFRA	6	4,8
PIK3CA	6	4,8
RET	6	4,8
MET	5	4,0
SMAD4	5	4,0
BRAF	4	3,2
ESR1	4	3,2
MTOR	4	3,2
NRAS	4	3,2
SF3B1	4	3,2
ALK	3	2,4
CTNNB1	3	2,4
IDH2	3	2,4
KIT	3	2,4

SMO	3	2,4
FGFR2	2	1,6
GNAQ	2	1,6
HRAS	2	1,6
AKT1	1	0,8
AR	1	0,8
ERBB3	1	0,8
GNAS	1	0,8
IDH1	1	0,8
PTEN	1	0,8

Supplementary Table S3. Relevant information regarding *NRAS* mutated patients.

	1300039	*13-003*	*13-027*	*36-005*
***NRAS* mutation by NGS (coding/protein)**	c.175G>C / p.A59P	c.37G>A / p.G13S	c.35G>A / p.G12D	c.181C>A / p.Q61K
**MAF *NRAS* mutation (%)**	1.89	0.24	0.53	1.80
***EGFR* mutation by NGS (coding/protein)**	Non detected	Non detected	c.2235_2249del15 / p.E746_A750del	c.2573T>G / p.L858R
**MAF *EGFR* mutation (%)**	-	-	1.98	25.63
**p.T790M by NGS**	Non detected	Non detected	Non detected	Non detected
***EGFR* mutation in tumor at stage IV diagnosis**	p.G719X	p.L861Q	p.E746_A750del	p.L858R
**Sex**	Male	Female	Female	Female
**Age (years)**	58	64	89	52
**Smoking**	Smoker	Former smoker	Never smoker	Never smoker
**Histology**	Adenocarcinoma	Adenocarcinoma	Adenocarcinoma	Adenocarcinoma
**ECOG-PS**	NA	0	1	1
**Metastases location at stage IV diagnosis**	Thoracic	Thoracic	Thoracic	CNS
**Stage**	IVA	IVA	IVA	IVB
**First-line TKI**	Afatinib	Gefitinib	Erlotinib	Afatinib
**PFS (months)**	16	9.7	6.6	7.6
**Progression sites**	NA	Thoracic	Thoracic	NA
**Second-line treatment**	Osimertinib	Chemotherapy	None	Osimertinib
**Exitus**	NA	0	1	1
**OS (months)**	NA	62.3	11.8	9.7

CNS: Central Nervous System; ECOG-PS: Eastern Cooperative Oncology Group Performance Status; MAF: Mutant Allele Frequency; NA: Not Available; NGS: Next-Generation Sequencing; OS: Overall survival; PFS: Progression-Free Survival.

Supplementary Table S4. Relevant information regarding *HRAS* mutated patients.

	400003	*15-003*
***HRAS* mutation by NGS (coding/protein)**	c.37G>C / p.G13R	c.183G>T / p.Q61H
**MAF *HRAS* mutation (%)**	4.07	0.40
***EGFR* mutation by NGS (coding/protein)**	Non detected	c.2236_2250del15 / p.E746_A750del
**MAF *EGFR* mutation (%)**	-	13.80
**p.T790M by NGS**	Non detected	Detected
***EGFR* mutation in tumor at stage IV diagnosis**	p.G719X	p.E746_A750del

**Sex**	Male	Male
**Age (years)**	74	73
**Smoking**	Former smoker	Never smoker
**Histology**	Adenocarcinoma	Adenocarcinoma
**ECOG-PS**	NA	1
**Metastases location at stage IV diagnosis**	Thoracic and bone	Bone
**Stage**	IVB	IVB
**First-line TKI**	Afatinib	Erlotinib
**PFS (months)**	5.8	17.1
**Progression sites**	NA	Bone
**Second-line treatment**	None	Osimertinib
**Exitus**	NA	No
**OS (months)**	NA	53.4

ECOG-PS: Eastern Cooperative Oncology Group Performance Status; MAF: Mutant Allele Frequency; NA: Not Available; NGS: Next-Generation Sequencing; OS: Overall survival; PFS: Progression-Free Survival.
